# Comprehensive analysis of B3 family genes in pearl millet (*Pennisetum glaucum*) and the negative regulator role of *PgRAV-04* in drought tolerance

**DOI:** 10.3389/fpls.2024.1400301

**Published:** 2024-07-29

**Authors:** Yin-Hua Wang, Xing Ye, Bi-Yao Zhao, Wen-Jing Wang, Zheng-Feng Zhou, Xiang-Qian Zhang, Juan Du, Jian-Ling Song, Xiao-Ling Huang, Kun-Xi Ouyang, Tian-Xiu Zhong, Fei-Xiong Liao

**Affiliations:** ^1^ Department of Grassland Science, College of Forestry and Landscape Architecture, South China Agricultural University, Guangzhou, China; ^2^ Guangdong Engineering Research Center for Grassland Science, Guangzhou, China; ^3^ College of Food Science and Engineering, Foshan University, Foshan, China; ^4^ Institute for Agricultural Biosciences, Oklahoma State University, Ardmore, OK, United States; ^5^ College of biology and chemistry, Minzu Normal University of Xingyi, Xingyi, China

**Keywords:** B3 superfamily, drought tolerance, gene expression, pearl millet, *PgRAV-04*

## Abstract

**Introduction:**

Members of the plant-specific B3 transcription factor superfamily play crucial roles in various plant growth and developmental processes. Despite numerous valuable studies on B3 genes in other species, little is known about the B3 superfamily in pearl millet.

**Methods and results:**

Here, through comparative genomic analysis, we identified 70 B3 proteins in pearl millet and categorized them into four subfamilies based on phylogenetic affiliations: ARF, RAV, LAV, and REM. We also mapped the chromosomal locations of these proteins and analyzed their gene structures, conserved motifs, and gene duplication events, providing new insights into their potential functional interactions. Using transcriptomic sequencing and real-time quantitative PCR, we determined that most *PgB3* genes exhibit upregulated expression under drought and high-temperature stresses, indicating their involvement in stress response regulation. To delve deeper into the abiotic stress roles of the B3 family, we focused on a specific gene within the RAV subfamily, *PgRAV-04*, cloning it and overexpressing it in tobacco. *PgRAV-04* overexpression led to increased drought sensitivity in the transgenic plants due to decreased proline levels and peroxidase activity.

**Discussion:**

This study not only adds to the existing body of knowledge on the B3 family’s characteristics but also advances our functional understanding of the *PgB3* genes in pearl millet, reinforcing the significance of these factors in stress adaptation mechanisms.

## Introduction

Pearl millet (*Pennisetum glaucum*), an annual herb native to Africa that grows mainly in arid and semi-arid tropical regions, is the world’s sixth-largest cereal crop (Sun et al., 2020). It has excellent tolerance to environmental stresses, especially high-temperature and drought stresses, and can be grown in areas where other crops cannot survive; it is thus considered an ideal crop for studying plant resilience and has attracted attention since its genome was sequenced (Swaminathan et al., 2008; Varshney et al., 2017; Dudhate et al., 2018; Chanwala et al., 2020; Huang et al., 2021). Transcription factors (TFs) are key regulators that play important roles in the endogenous defense systems of plants in a variety of stress responses. Therefore, the analysis of gene changes in pearl millet under abiotic stress provides a practical basis for cereal crop research for the benefit of modern agriculture.

The B3 family is a class of DNA-binding TFs with a highly conserved domain unique to plants and plays a key role in many aspects of plant development. It is named after its B3 DNA-binding domain; the conserved B3 domain includes approximately 110 amino acid residues for DNA recognition, consisting of seven β-barrels and two short α-helices (Kagaya et al., 1999; Swaminathan et al., 2008). The B3 superfamily is divided into four families: ARF (auxin response factor), LAV (leaf cotyledon2-abscisic acid insensitive3-VAL), RAV (related to ABI3/VP1), and REM (reproductive meristem) (Swaminathan et al., 2008; Sasnauskas et al., 2018). In addition, B3 TFs play important roles in plant adaptation and survival under various abiotic and biotic stresses (Verma and Bhatia, 2019; Ren et al., 2023). Since its discovery, the B3 family has been characterized in many model plants and crops including Arabidopsis, rice, poplar, rapeseed, castor, soybean, tobacco, and grape (Ahmad et al., 2019; Xia et al., 2019; Liu et al., 2020; Wang et al., 2022a; Wei et al., 2023) but not yet in pearl millet.

As a member of the B3 family, RAVs play important roles in developing plants and defense against abiotic stresses. Ectopic expression of soybean *GmRAV3* in Arabidopsis can improve the resistance of transgenic lines to high salt and drought conditions and lead to the insensitivity of transgenic plants to exogenous abscisic acid (Zhao et al., 2017). Melon *CmRAV1* can be induced by NaCl treatment, and ectopic overexpression of *CmRAV1* in Arabidopsis enhances salt tolerance during seed germination and growth (Zhao et al., 2019). According to recent research, ABA and NaCl treatments affect cucumber *RAV1*, while ectopic expression of *CsRAV1* in Arabidopsis enhances its tolerance to NaCl and ABA (Li et al., 2023). Investigations into the function of the wheat *TaRAV1* gene revealed that overexpressing *TaRAV1* improved the ability of Arabidopsis to tolerate salt (Luo et al., 2022). RAV TFs were also associated with anthocyanin synthesis, further influencing abiotic stress in plants, as demonstrated in strawberries and pears (Zhang et al., 2020b; Liu et al., 2021b).

Pearl millet exhibits excellent tolerance to environmental stresses, particularly to high temperature and drought stress. Among the many regulatory factors in plant growth and development, members of the B3 family play an extremely important role and exhibit unique gene functions among different species. Therefore, research on the B3 family genes in pearl millet can not only deepen the understanding of *B3* genes functions but also fill existing knowledge gaps, providing a theoretical basis and practical methods for future research on plant tolerance. We utilized bioinformatics methods to investigate the physicochemical properties, structural characteristics, phylogenetic evolution, chromosomal localization, and gene duplication of the B3 family in pearl millet. We identified a total of 70 B3 family genes that were classified into four subfamilies (ARF, REM, RAV, and LAV) by phylogenetic analysis. These 70 *PgB3* genes are distributed across seven chromosomes and were observed to have undergone purifying selection during the evolutionary process. In addition, transcriptomic data and real-time fluorescence quantification analyses revealed that most genes in the B3 family responded to drought and high-temperature stresses. Among these genes, *PgRAV-04* expression decreased under drought treatment but increased briefly under high temperature treatment. Therefore, we cloned *PgRAV-04* and ectopically overexpressed it in *Nicotiana benthamiana* to study the function of this gene. Overexpression of the *PgRAV-04* gene in tobacco led to an increase in malondialdehyde (MDA) content and decreases in proline content, relative leaf water content, and antioxidant enzyme activity, thereby increasing the plant’s sensitivity to drought. In conclusion, *PgRAV-04* acts as a negative regulatory factor in response to drought stress.

## Materials and methods

### Database research and sequence retrieval

Pearl millet genome and protein sequences were downloaded from the GigaDB database (http://gigadb.org/dataset/100192); the pearl millet protein data set was built using the TMHMM server 2.0 (http://www.cbs.dtu.dk/services/TMHMM/). In addition, the hidden Markov model (HMM) profile of the B3 domain (PF02362) was downloaded from the Pfam database (Pfam; http://pfam.xfam.org/) (Finn et al., 2014) to be utilized for the identification of B3 genes from the local database (E-value < 0.001) using HMMER. All hits were confirmed by Pfam (PF02362) and NCBI conserved domain searches (http://www.ncbi.nlm.nih.gov/Structure/cdd/wrpsb.cgi/). The SMART database (http://smart.embl-heidelberg.de/smart) and ExPASy-PROSITE database (http://www.expasy.org/prosite) were used to further confirm the presence of the B3 domain in all members of the B3 superfamily. The confirmed B3 protein sequences were aligned using Clustal X (v 2.0 http://www.clustal.org/clustal2/) to remove redundant sequences, then sequences with a confidence level > 97% and an alignment length > 50% were retained. The molecular weights and theoretical isoelectric points of all candidate B3 proteins were calculated using the ExPASy program (https://web.expasy.org/protparam/). The B3 protein sequences of Arabidopsis were downloaded from The Arabidopsis Information Resource (https://www.arabidopsis.org/); rice B3 protein sequences were retrieved from the Rice Genome Annotation Project (http://rice.plantbiology.msu.edu/).

### Phylogenetic tree, motif, and gene structure analyses

A total of 70 B3 protein sequences from pearl millet were aligned using Clustal X (v2.0, http://www.clustal.org/clustal2/). Two neighbor-joining phylogenetic trees were constructed and visualized using the MEGA-X software with 1000 bootstrap replications: one for pearl millet and another for pearl millet, rice, and Arabidopsis. The conserved motifs of the B3 proteins were identified using MEME v5.0.5 (http://meme-suite.org) (Bailey et al., 2009) with the following parameters: the maximum number of motifs was set to 10 for the prediction of subdomains; the motif width was set to between 6 and 100. Motif analysis was carried out using the TBtools visualize domain pattern. The exon–intron substructure map of each B3 gene was generated according to the Gene Structure Display Server (GSDS 2.0, http://gsds.cbi.pku.edu.cn/) (Hu et al., 2015).

### Chromosome location and gene duplication events

The chromosome location images of *PgB3* genes were generated using the MapInspect software (http://mapinspect.apponic.com/) (Ma et al., 2013) according to the available information from the genome database. The MCScanX (http://chibba.pgml.uga.edu/mcscan2/) software was used to estimate gene duplication event types in the pearl millet B3 superfamily genes. Tandem gene duplication in pearl millet was defined as paralogous genes physically linked in tandem with less than five genes and/or genes located within 100 kb of each other that belonged to the same family (Zhang et al., 2020a; Liu et al., 2021a). Segmental duplications were those placed on replicated chromosomal blocks from the same genome lineage that isolated more than five genes. Tandem duplications and segmental duplications were visualized using TBtools software. The synonymous substitution rate (Ks) and nonsynonymous substitution rate (Ka) of duplicated genes were obtained using the online tool kaks_calculator2.0 (https://sourceforge.net/projects/kakscalculator2/) (Gao et al., 2015). The Ka/Ks ratio is commonly used to understand the direction of evolution and can reveal three different situations: Ka/Ks > 1 indicates a positive selection, Ka/Ks < 1 indicates a negative selection, and Ka/Ks = 1 indicates a neutral selection (Li et al., 2009; Wang et al., 2009).

### Prediction of the PgB3 protein–protein interaction network

An interaction network of PgB3 proteins was built to retrieve all possible interactions based on orthologous Arabidopsis proteins using the STRING 10 software (https://string-db.org/). Preprocessing of the interaction network included removal of redundancy and self-looping interactions, then target proteins with credibility > 0.7 were selected to construct a regulation network via the OmicStudio tools (https://www.omicstudio.cn/tool); the nodes represent the protein/gene, and the lines indicate the interactions between two proteins/genes.

### Plant material and stress treatments

In an artificial climate chamber maintained at 30°C/24° (day/night) with a 16h/8h (day/night) photoperiod, the relative humidity of 50%-75%, and then pearl millet seeds were planted in 1-gallon pots containing a mixture of substrate and vermiculite (4:1, v/v); experiments were repeated in triplicate using a random block design. For stress response analysis, 5-week-old seedlings were treated with high temperature of 42°C/35°C (day/night) with a photoperiod of 16/8h (day/night), the relative humidity of 50%-75%, and drought (non-irrigation) for a specified period, followed by a recovery period. Plant leaves were sampled at different time points under drought (0h, 12h, and 48h, then after 2 days of recovery) and high temperature (0h, 12h, and 148h, then after 2 days of recovery) treatments; these samples were used for transcriptome analysis. Under the same stress treatments, we performed real-time fluorescence quantification experiments for both drought and high temperature at 0h, 12h, and 48h, then after 2 days of recovery. All samples were taken from three replicates and immediately stored at −80° for RNA extraction; the leaves sampled at 0h served as controls for the stress treatments.

### Expression profiling of *PgB3* genes by RNA-seq and qRT-PCR

To determine the expression profiles of the *PgB3* genes under drought and high-temperature stresses, samples from the different stress treatments were subjected to transcriptome sequencing. All samples were stored on dry ice and sent to LC-Bio Technologies (Hangzhou, China) for RNA extraction and sequencing. To identify the differentially expressed genes that respond to drought and high temperature stress, we screened them using the criteria |log_2_FC| > =1 and q<0.05. Mapped reads were used to measure the fragments per kilobase of transcript per million mapped reads (FPKM) values; heat maps were generated using log_2_ Z-score values in the OmicStudio tools (https://www.omicstudio.cn/tool) and clustering in heat maps was based on correlation.

For qRT-PCR analyses, total RNA was extracted using TRIzol reagent (TianGen, Beijing, China) and first-strand cDNA was synthesized with a reverse transcription kit (Transgen, Beijing, China). Six gene-specific primer pairs were designed using the Primer Premier5.0 software (Premier Biosoft Intl, California, USA) (**Supplementary Table S1**). qRT-PCR was performed on the LightCycler 480 Real-Time PCR System using the PerfectStart Green qPCR Supermix reaction kit (Transgen, Beijing, China): 10 µL reactions contained 5 µL 2× PerfectStart Green qPCR Supermix, 0.2 µL each of forward and reverse primer (10.0 µmol/L), 0.5 µL cDNA (5-fold diluted), and 4.1 µL RNase-free water. qRT-PCR amplification was carried out as follows: 94° for 30s, followed by 40 cycles of 94° for 5s and 60° for 30s. The pearl millet *EF-1ɑ* gene (Shivhare and Lata, 2016) was used as the reference gene to normalize the qRT-PCR data; relative gene expression levels were determined via the 2^−ΔΔCT^ method. Each experiment was performed using three biological replicates.

### Gene cloning and transformation of *PgRAV-04* into tobacco

The *PgRAV-04* gene, involved in the response to drought and high-temperature stresses, was selected to confirm gene functions. *PgRAV-04* transgenic lines were generated by cloning the coding sequence of *PgRAV-04* into the pUbi-intron-OE vector (preserved in this laboratory) between the *Spe* I and *Bam*H I restriction sites using the primers listed in **Supplementary Table S1**; the expression vector Ubi: *PgRAV-04* was transformed into *Agrobacterium tumefaciens* (EHA105), followed by *N. benthamiana* transformation using the leaf disc method.

### Molecular confirmation of transgenic tobacco plants

Genomic DNA was isolated from the leaves of putative transgenic and wild type plants. The presence of *PgRAV-04* in the transgenic plants was confirmed by PCR analysis using gene-specific primers (**Supplementary Table S1**); WT plants were used as negative controls, and plasmids containing fragments of interest were used as positive controls. The amplified products were electrophoresed on a 1% (w/v) agarose gel. PCR-positive plants were further confirmed by qRT-PCR. RNA was isolated and cDNA was synthesized as described above. The tobacco *Actin* gene was selected as a reference gene for expression in transgenic tobacco (**Supplementary Table S1**).

### Physiological and biochemical indices under stress treatments

For stress response analysis of transgenic tobacco, transgenic plants were planted in 1-gallon pots containing a mixture of substrate and vermiculite (4:1, v/v). The plants were grown in a growth chamber under 16h light (100 μmol m^−2^ s^−1^) and 8h dark; humidity was maintained at 60-70% and the temperature was controlled at 28°C/25°C (day/night). Five-week-old *PgRAV-04* transgenic tobacco plants were used for drought analysis, it is worth noting that the WT (wild type) material did not undergo transfer of the target gene, nonetheless, the seedlings used in the experiment were obtained through the same tissue culture process that was employed for the transgenic tobacco. In this study, we assessed the drought tolerance of three transgenic tobacco lines compared to the WT plants. Physical indices were determined on days 0d, 9d, and 16d of drought treatment and after 3 days of rehydration; each sample had three biological replicates. Leaf relative water content (RWC), malondialdehyde content (MDA), proline content, superoxide dismutase activity (SOD), peroxidase activity (POD), ascorbate peroxidase activity (APX), and catalase activity (CAT) were determined following previously described protocols (Zhong et al., 2014).

### Date statistical analysis

The SPSS software was utilized for significance analysis. Real-time fluorescent quantitative analysis utilized Duncan’s multiple range test while physiological indicators were assessed using independent sample *t*-tests, with significance levels set at p < 0.05 and p < 0.01 to evaluate the differences among various treatments. The data are presented as mean values with the standard error of the mean for three replicates.

## Results

### Identification and analysis of pearl millet B3 family genes

After searching the pearl millet genome, a total of 70 B3 family genes containing the plant-specific B3 domain were identified. Based on other domains in the protein sequences, the *PgB3* genes were classified into four subgroups: LAV (5 genes), RAV (10), ARF (22), and REM (33) (**Table 1**). The gene lengths ranged from 475 bp (*PgRAV_03*) to 3183 bp (*PgARF_17*). The amino acid lengths of encoded proteins ranged from 154 to 1039 aa, among which three proteins (PgARF_14, PgARF_17 and PgARF_18) were >1000 aa in length and 32 proteins were >500 aa in length; molecular weights ranged from 17.374 kDa to 140.283 kDa and isoelectric points were between 4.95 and 10.14. In addition, 20 gene members contained more than 10 exons while 4 gene members (*PgRAV _ 02*, *PgRAV _ 03*, *PgRAV _ 04*, and *PgRAV _ 06*) had only one exon (**Supplementary Table S2**).

**Table 1:** Comparison the B3 family of pearl millet with Arabidopsis and rice.

Spescies	LAV	RAV	ARF	REM	Total
*Arabidopsis*	6(5.08%)	13(11.01%)	23(19.49%)	76(64.41%)	118
Rice	7(7.69%)	16(17.58%)	28(30.77%)	40(43.96%)	91
Pearl millet	5(7.14%)	10(14.29%)	22(31.43%)	33(47.14%)	70

### Phylogenetic and structural analyses of pearl millet B3 family genes

**Figure 1:**
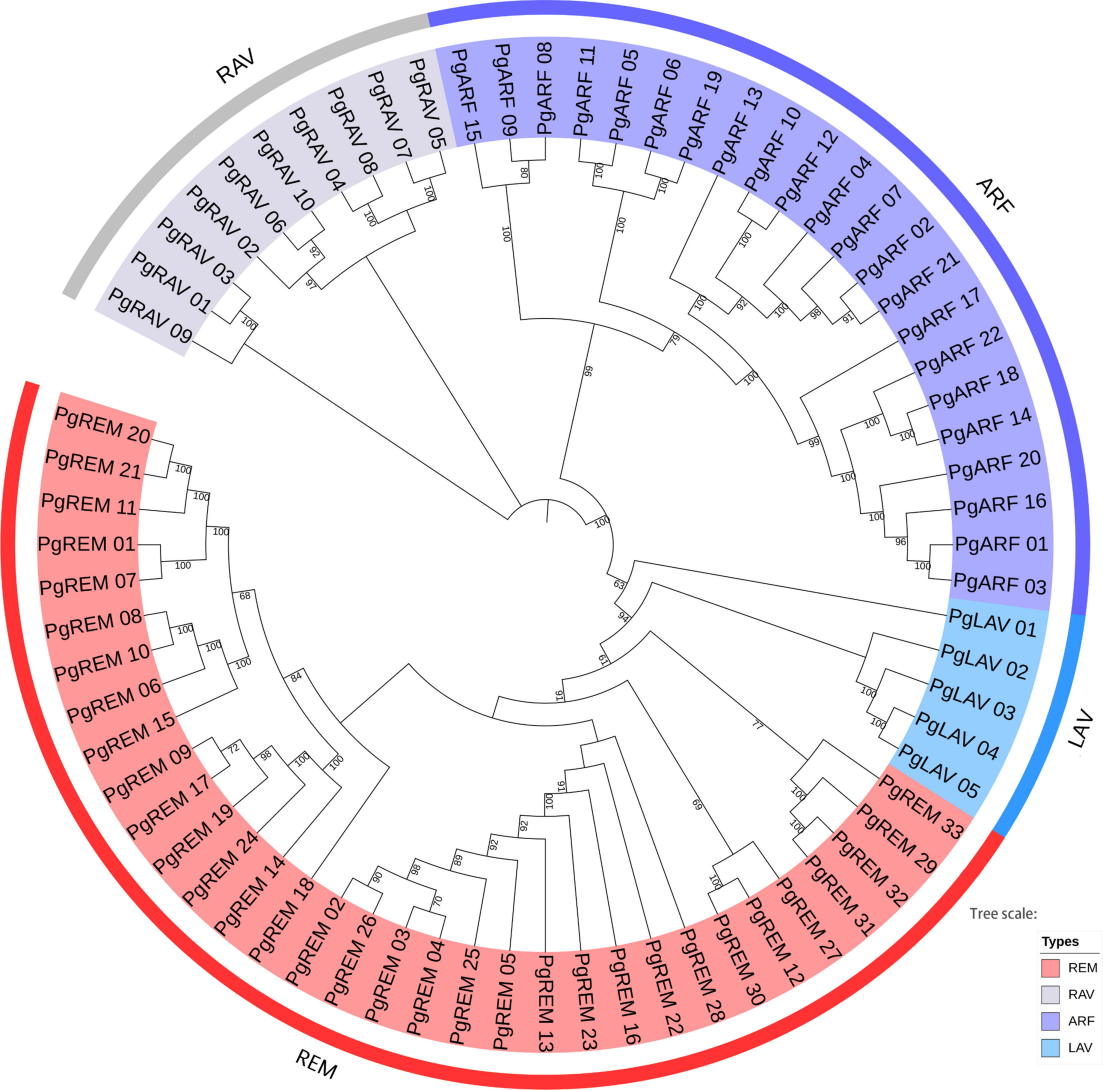
Phylogenetic tree of pearl millet B3 family genes. The neighbor-joining tree is divided into four groups (REM, LAV, ARF, and RAV). The different colored areas indicate different groups of the B3 superfamily.

To examine the phylogenetic relationships of the B3 gene family in pearl millet and to assist in their classification, we built a neighbor-joining phylogenetic tree. According to the clade support values, the B3 genes were divided into four distinct subfamilies: LAV, RAV, ARF, and REM (**Figure 1**). The REM subfamily had the most *PgB3* genes (33), followed by ARF (22), while the LAV subfamily had the fewest *PgB3* genes. In addition, another phylogenetic tree was constructed with the 70 *PgB3* genes as well as B3 family genes from other species to confirm the phylogenetic relationships throughout the evolutionary process (**Supplementary Figure S1**); most results were similar using both approaches except that the rice *RAV* (*Os07g17230*) gene was clustered into the REM subfamily. Furthermore, according to the constructed phylogenetic trees and a review of the literature, we were able to pinpoint functionally annotated B3s with their *PgB3* homologs (**Supplementary Table S3**); in all, 34 *PgB3* homologous genes were predicted, indicating that the pearl millet B3 superfamily genes were highly conserved during the course of evolution.

### Gene structure and conserved motif analyses of pearl millet B3 family genes

**Figure 2:**
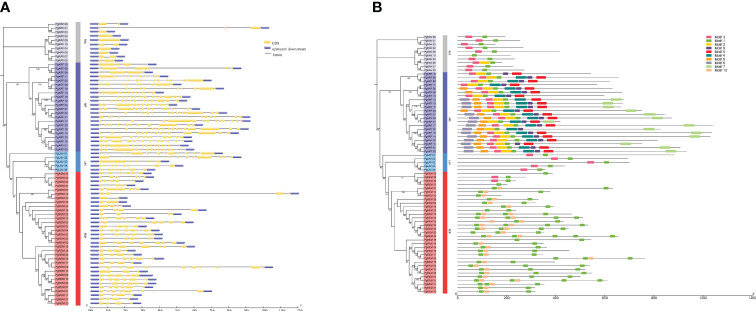
Gene structure and conserved motif analyses of PgB3s. **(A)** Gene structural analysis was performed according to the phylogenetic tree. The exons, introns, and untranslated regions are represented by yellow boxes, black lines, and blue boxes, respectively. **(B)** Conserved motif analysis. Each motif is represented by a number of colored boxes; the length of the box indicates the motif length.

To understand the structural diversity of the pearl millet B3 family genes, we analyzed the organization of the exons and introns in the coding regions. The number of introns in *PgB3* genes ranged from 0 to 14 (**Figure 2A**), with ranges of 3-14, 5-10, 1-10, and 0-2 in the ARF, LAV, REM, and RAV groups, respectively. In general, most genes within each B3 subfamily had similar intron numbers, structures, and lengths, supporting the subgroup classification in the phylogenetic tree.

We also observed that the distribution of conserved motifs differed among subfamilies. Ten conserved motifs were identified in the PgB3 proteins (**Figure 2B**): motif 1 was the B3 DNA-binding domain, motifs 4 and 9 corresponded to auxin response factor domains, motif 7 was the Aux/IAA domain (**Table 2**), and the remaining six were not annotated. Conserved motif 1 was found in almost all members of the pearl millet B3 family; motifs 4 and 9 were found in all members of the ARF subfamily; motif 10 was only present in the REM subfamily; and the RAV, ARF, and LAV subgroups all had motif 3. These results indicate that possession of specific motifs may be associated with gene differentiation and diversity.

**Table 2:** Distribution and annotation of PgB3 conserved amino acid motifs.

NO.	E-value	Width	Location	Conserved motifs	Annotation
**1**	3.5e-859	21	115	GWSXFVX[ADE][NKH][RG]LX[AE]GDI[CLV][LV]FXR	B3 DNA binding domain: DNA binding
**2**	1.2e-589	41	22	PPLD[YFM]S[QM]QPPAQEL[VI]A[KR]DLH[GD]NEW[KR]FRHI[FY]RGQP[RK]RHLLTT	NO
**3**	5.4e-497	29	34	H[LS]FCKT[LV]T[AP]SD[TV][SG][TK][HL][GN][GR][FL][SV]VP[RK][RQ][HA]AEKC[FL]	NO
**4**	8.1e-379	46	18	[DHQ]SMH[IL]GVLA[AT][AV][AW]HA[AVI][ASN][TN][NGR][ST][MV]F[TH][IV][FY]Y[NK]PR[TA]SPSEF[VI][IV][PS][LY][AD][KR][YF][ML][KE][AS]L[YKN]	Auxin response factor: DNA binding
**5**	2.2e-320	40	20	WP[NGD]SKWRS[LV][KQ]V[RG]WD[ED][PS][ST]A[GI]ER[PQ][NDEP]RVS[PL]WE[IV]E[PL][LV][ST][SAT][SFP]P[MP][IY][PH]	NO
**6**	1.10E-306	41	14	[AC][LI][NFY]SELWHACAGPLV[ST][LV]P[PR]VG[SE][RLK]V[VY]YFPQGH[SI]EQ[VL][AE]AS[TM]N[KQ]	NO
**7**	1.60E-252	45	12	W[KQM][LV]V[YF][TV]D[NH]E[DGN]D[IM][LM]LVGDDPW[EQ]EF[VC][NS][MC]V[HK][CK]I[KF]I[LY][ST]P[EQ]EVQQM[NS][LP][DG]G	AUX/IAA domain: protein binding
**8**	3.60E-229	32	14	NLP[SP][KQ][LI][IL]C[QK][LV][HV]NV[TE][LM][HK]A[DE][AP][DE]TDEVYAQ[MIL][TM]LQP	NO
**9**	4.90E-192	22	20	[QNR][IY]S[VI]GMRF[KR]M[RL]FE[TG][ED][ED][SA][SP][EV][RQ][RS][YF]	Auxin response factor: DNA binding
**10**	3.50E-189	21	33	GN[SM]V[FL][TK]V[KL][IV]FD[AP][SD]GCE[KR][EV]F[KS]C	NO

The location is the motif number and distribution, the width is the width of the motif, and the E-value is the statistical significance of the motif.

### Chromosomal location and duplication events of pearl millet B3 family genes

**Figure 3:**
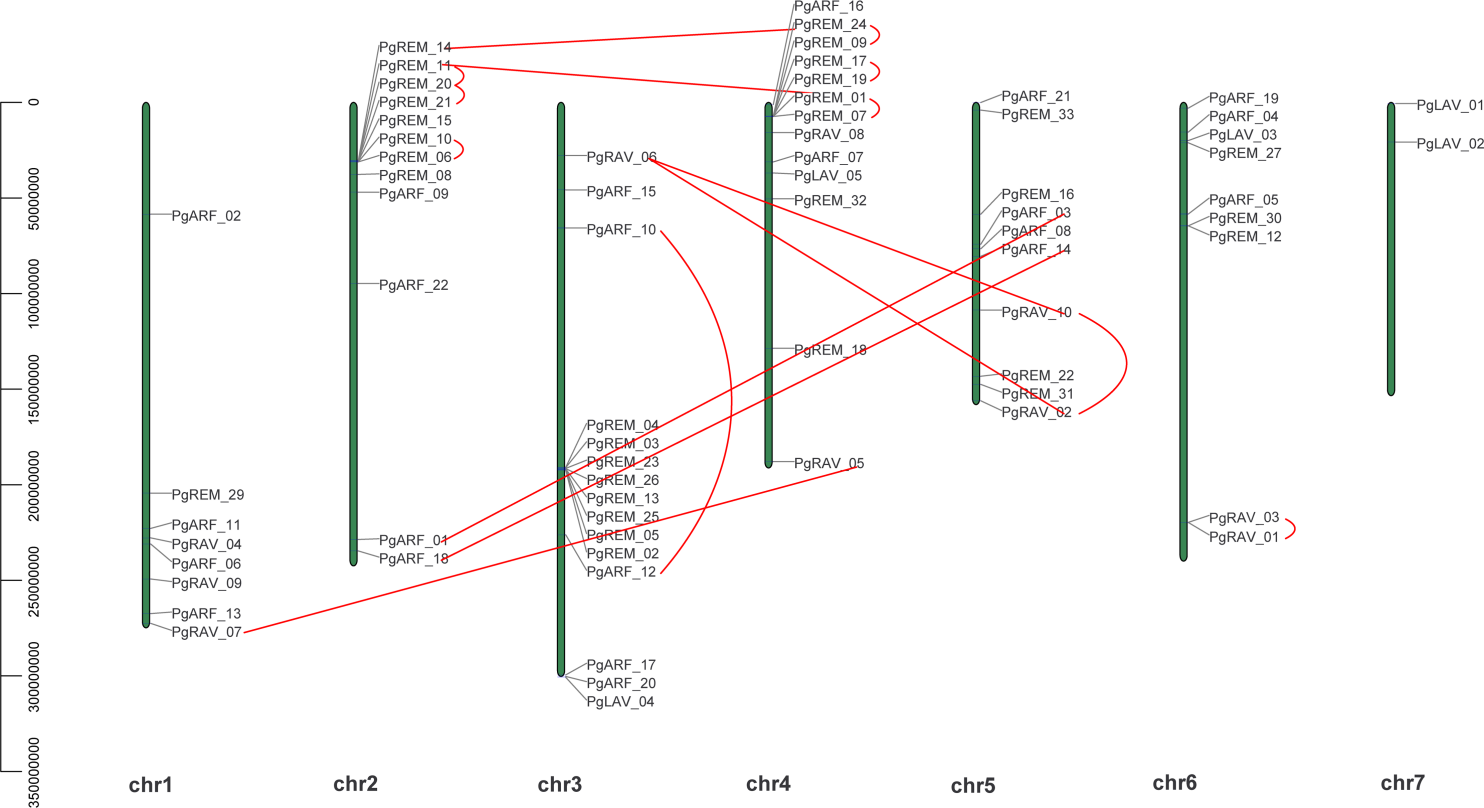
Chromosomal locations of *PgB3* genes. The position and full length of *PgB3* genes are indicated by the numbers on the left-hand side of the chromosome; the chromosome number is indicated below the chromosome. The segmental duplication is connected by the straight red line, while tandem duplications are connected by curved red lines.

Chromosomal distribution analysis revealed that 69 of the 70 *PgB3* genes were randomly distributed across the chromosomes (**Figure 3**), with the most on Chr3 (15) and the least on Chr7 (2); chromosomes 1, 2, 4, 5, and 6 contained 8, 12, 13, 10, and 9 members, respectively.

Gene duplications including tandem duplication (in which the duplication segment is contiguous with the original duplication), segmental duplication (the copying of whole blocks of genes from one chromosome to another chromosome) (Leister, 2004), and whole-genome duplication (characterized by an organism’s entire genetic information being copied one or more times) (Lee et al., 2020) may play important roles not only in the process of genome rearrangement and expansion but also in the process of diversification of gene functions and generation of gene families. In this study, a survey of gene duplication analyses indicated that nine gene pairs (17 genes) formed segmental duplication events and seven gene pairs (13 genes) were involved in tandem duplications (**Figure 3**, **Table 3**), the number of segmental and tandem duplications was similar, suggesting that both types of gene duplication promote amplification of *PgB3* family genes.

**Table 3:** Evolutionary selection types of the *PgB3* genes.

Gene pair	Chromosomal localization	Ka	Ks	Ka/Ks	Evolutionary type	Duplication type
PgREM_11-PgREM_01	chr2/chr4	0.6668	1.65072	0.403945	Purifying	Segmental
PgREM_14-PgREM_24	chr2/chr4	0.585942	2.42752	0.241374	Purifying	Segmental
PgRAV_06-PgRAV_02	chr3/chr5	0.420515	0.603796	0.696452	Purifying	Segmental
PgRAV_10-PgRAV_02	chr5/chr5	0.510665	0.58405	0.874351	Purifying	Segmental
PgRAV_05-PgRAV_07	chr4/chr1	0.326856	1.04242	0.313555	Purifying	Segmental
PgRAV_10-PgRAV_06	chr5/chr3	0.336143	0.779097	0.431452	Purifying	Segmental
PgARF_01-PgARF_03	chr2/chr5	0.125456	0.860205	0.145844	Purifying	Segmental
PgARF_10-PgARF_12	chr3/chr3	0.19923	1.04497	0.190655	Purifying	Segmental
PgARF_18-PgARF_14	chr2/chr5	0.137527	0.948927	0.144929	Purifying	Segmental
PgREM_20-PgREM_21	chr2/chr2	0.104597	0.154981	0.674902	Purifying	Tandem
PgREM_11-PgREM_20	chr2/chr2	0.280939	0.493584	0.569181	Purifying	Tandem
PgREM_10-PgREM_06	chr2/chr2	0.256997	0.665422	0.386217	Purifying	Tandem
PgRAV_03-PgRAV_01	chr6/chr6	0.198966	0.58306	0.341245	Purifying	Tandem
PgREM_01-PgREM_07	chr4/chr4	0.311148	0.881141	0.353119	Purifying	Tandem
PgREM_17-PgREM_19	chr4/chr4	0.25588	0.60614	0.422146	Purifying	Tandem
PgREM_24-PgREM_09	chr4/chr4	0.350261	0.874533	0.400512	Purifying	Tandem

Ka, non-synonymous substitution rate; Ks, synonymous substitution rate; Ka/Ks > 1 indicates a positive selection, Ka/Ks < 1 indicates a negative selection, and Ka/Ks = 1 indicates a neutral selection.

Purifying selection preserves the innate adaptability of species, whereas diversifying selection supports evolutionary innovation (beneficial mutations) and species differentiation (Yan et al., 2017). To understand the evolutionary trends of these *PgB3* genes, detailed information on Ka, Ks, and Ka/Ks is presented in **Table 3**. The Ka/Ks values of all tandemly repeated *PgB3* sequences were > 1, implying that they were all targets of purifying selection during the evolutionary process.

### Analysis of the PgB3 protein interaction network

**Figure 4:**
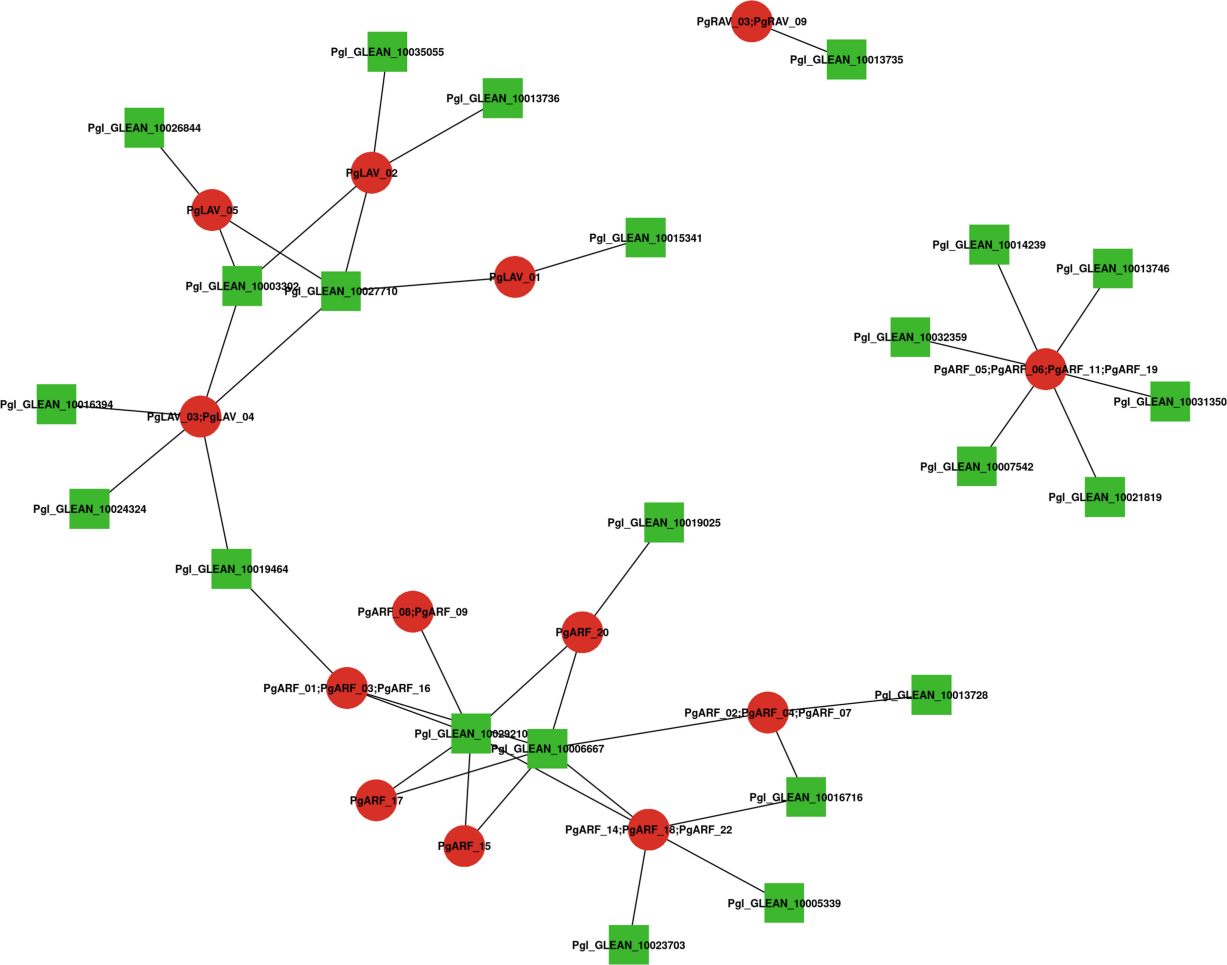
Interaction network analysis of B3 proteins identified in pearl millet according to their orthologs in Arabidopsis. Nodes represent proteins and lines represent interactions between two proteins; red represents PgB3 family proteins, green represents other proteins in pearl millet.

A network of integrated proteins was built using Arabidopsis homologs to further investigate the relationships between PgB3 proteins (**Supplementary Table S4**). Among the PgB3 proteins, a total of 25 interacting proteins were identified with high confidence (**Figure 4**); when combined with the gene annotation results (**Supplementary Table S3**), a total of 18 of the interacting proteins had predicted functions. The homolog of PgARF_05/06/11/19 is AT2G33860.1, which had the highest number of proteins with direct interactions in the interaction network (six proteins), followed by PgLAV_03/04 and PgARF_14/18/22 interacting with five proteins. Notably, many proteins are involved in stress and disease resistance, for example: PgARF_01/03/06, PgARF_02, PgARF_07, PgARF_14/16, PgARF_14/20, and PgARF_19. In addition, PgARF_17 and PgARF_14/18 were associated with the auxin signaling pathway.

### Expression profiles of *PgB3* genes under drought and high-temperature stresses

**Figure 5:**
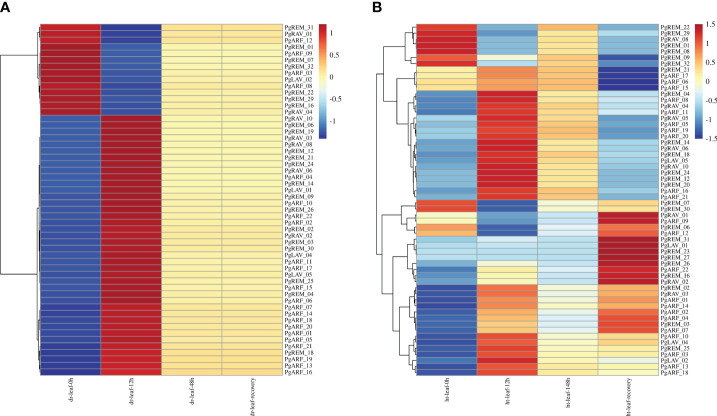
Expression levels of *PgB3* gene transcripts in pearl millet in response to abiotic stresses. **(A)** Drought stress at different treatment times (0h, 12h, 48h and after 2 days of recovery). **(B)** High-temperature stress at different treatment times (0h, 12h, 148h and after 2 days of recovery). The color scale represents increased (red) and decreased (blue) fold-changes in the levels of *PgB3* transcripts at different treatment times when exposed to abiotic stress (dr: drought; ht: high temperature) compared to the respective levels in the controls for each stress. The fold-change is shown on a log_2_ scale from high to low expression of each *PgB3* transcript.

Because the B3 family plays a crucial role in how plants respond to abiotic stresses, we used RNA-seq data from pearl millet under high-temperature and drought treatments to further our understanding of the biological processes involving the *PgB3* genes (**Figure 5**). This analysis demonstrated that 54 *PgB3* genes responded to drought stress (**Figure 5A**; **Supplementary Table S5**) and 58 *PgB3* genes responded to high temperature (**Figure 5B**; **Supplementary Table S6**). Several genes were upregulated under both drought and high-temperature stresses, such as *PgARF_21*, *PgARF_05*, *PgARF_20*, *PgRAV_10*, and *PgREM_14*. Interestingly, *PgRAV_04*, *PgLAV_02*, and *PgARF_08* were downregulated by drought stress but upregulated by high-temperature stress. These results suggest that while many PgB3s have roles in drought and high-temperature stress responses, they may be functionally distinct.

**Figure 6:**
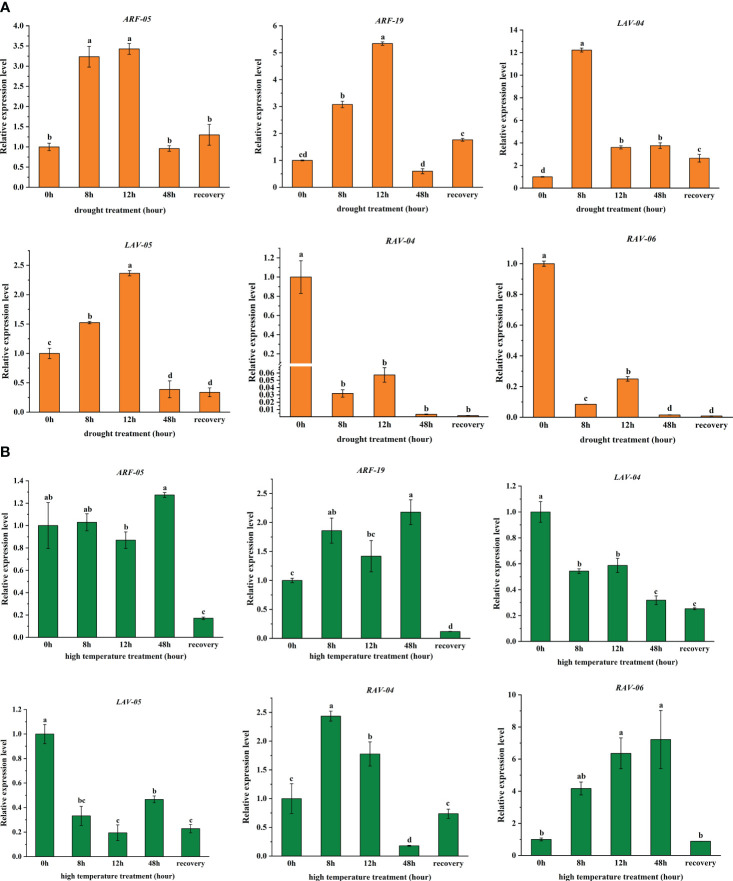
Expression analysis of six selected *PgB3* genes based on transcriptome data relating to **(A)** Drought and **(B)** High-temperature stresses through qRT-PCR. Relative expression of the *PgB3* genes was normalized relative to the reference gene *PgEF-1ɑ* under different stresses. Significant differences were determined according to Duncan’s multiple tests; data are means (± SDs) of triplicates; bars with different letters were significantly different (*P* < 0.05).

To validate the RNA-seq data under drought and high-temperature stress treatments (**Figure 5**), we characterized the expression of six *PgB3* genes using qRT-PCR. We found that all six genes responded to drought stress: while four genes (*PgARF_05*, *PgARF_19*, *PgLAV_04*, and *Pg LAV_05*) were upregulated in the early stages of drought, *PgRAV-04* and *PgRAV-06* were significantly downregulated (**Figure 6A**). *PgLAV_04* and *PgLAV_05* were downregulated under high-temperature stress, while *PgARF_05*, *PgARF_19*, *PgRAV-04*, and *PgRAV-06* were upregulated (**Figure 6B**). Notably, the expression levels of *PgRAV_04* and *PgRAV_06* were significantly low at 8h and 12h of drought treatment but were higher at 8h and 12h of high-temperature treatment. By contrast, expression levels of *PgLAV_04* and *PgLAV_05* were upregulated under drought stress and downregulated under high-temperature stress; however, *PgARF_05* and *PgARF_19* were upregulated under both drought and high-temperature stresses (**Figures 5**, **6**). These results support the expression profiles of the transcriptome and demonstrate the validity of the RNA-seq data. To confirm the role of *PgRAV_04* in abiotic stress responses, we used the *PgRAV_04* OE transgenic line for further analyses.

### Overexpression of the *PgRAV-04* gene resulted in drought-sensitive transgenic tobacco

**Figure 7:**
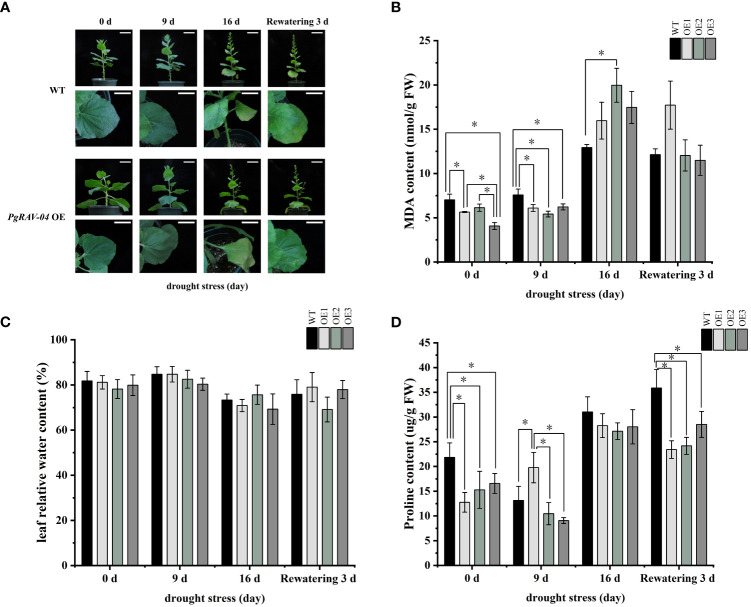
Effect of *PgRAV-04* overexpression (OEs) on tobacco drought tolerance during different periods of drought treatment. **(A)** Phenotypes of wild-type (WT) and the three *PgRAV-04* OE lines during drought treatment and after 3 days of recovery. **(B)** Malondialdehyde content (MDA), **(C)** Leaf relative water content (RWC), and **(D)** Proline content in WT and the three *PgRAV-04* OE lines during drought stress and after 3 days of recovery. Data of the three transgenic lines are presented as means (± SDs) of triplicates, while the WT is represented by the average value of 9 repetitions. Significant differences between WT and transgenic lines were determined using independent samples *t*-tests. *P < 0.05.

We confirmed the successful transformation of *PgRAV-04* in transgenic plants by PCR and qRT-PCR (**Supplementary Figures S2**, **S3**). To investigate the function of the *PgRAV_04* gene in plant growth and development under drought stress, we subjected 5-week-old transgenic and WT plants to drought treatment (**Figure 7**). The transgenic plants showed severe wilting symptoms under severe stress (16 days of drought treatment) compared to WT plants (**Figure 7A**). After 16 days without irrigation, the MDA content in the three overexpression lines were higher than in the WT plants. Among them, the MDA content of OE2 is particularly notable, being 1.51 times that of the WT plants (**Figure 7B**; **Supplementary Table S7**). The relative water content of the three transgenic lines were generally lower than the WT plants at all stages of the drought treatment (**Figure 7C**; **Supplementary Table S7**). The proline content of the three transgenic tobaccos were significantly lower than the WT at 0 day, suggesting that our transgenic materials were inherently intolerant to osmotic stress; meanwhile, the proline content of the three transgenic plants were similarly lower than in the WT plants as drought increased (**Figure 7D**; **Supplementary Table S7**), further indicating that the *PgRAV-04* overexpression plants were sensitive to drought. In summary, the transgenic plants had higher MDA levels, lower RWC and proline levels, and a more pronounced wilt phenotype, suggesting that the transgenic plants were more sensitive to drought.

**Figure 8:**
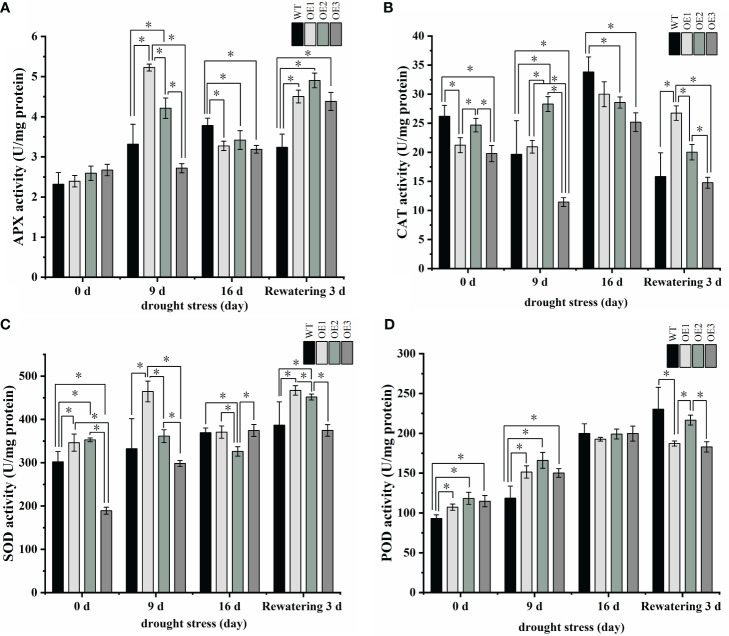
Antioxidant enzyme activity in WT and the three *PgRAV-04* OE lines. **(A)** Ascorbate peroxidase (APX), **(B)** Catalase (CAT), **(C)** Superoxide dismutase (SOD), and **(D)** Peroxidase (POD) activities in the leaves of WT and the three *PgRAV-04* OE lines under drought stress and after 3 days of recovery. Data of the three transgenic lines are presented as means (± SDs) of triplicates, while the WT is represented by the average value of 9 repetitions. Significant differences between WT and transgenic lines were determined using independent samples *t*-tests. *P < 0.05.

Because the MDA content was higher in the transgenic plants, which would imply membrane lipid peroxidation, we measured peroxidase activity, which quenches toxic free radicals. APX activities were initially higher in the three transgenic plants than in the WT plants but were significantly lower in the three transgenic plants compared to the WT after 16 days of drought (**Figure 8A**; **Supplementary Table S7**). At the same time, after 16 days of drought treatment, CAT, SOD, and POD activities were lower in the transgenic plants than in the WT plants (**Figures 8B–D**; **Supplementary Table S7**). In a word, these antioxidant enzyme activity results further suggest that the transgenic plants were more sensitive to drought. Altogether, we conclude that *PgRVA-04* strongly responds to drought but acts as a negative regulator under drought stress.

## Discussion

### Evolutionary diversity in the pearl millet B3 superfamily

The B3 superfamily has been extensively studied in various plants due to its diverse roles in vegetative and reproductive development. However, this study is the first report of a genome-wide analysis of the B3 superfamily in pearl millet, identifying a total of 70 B3 superfamily genes. This number is lower than those found in Arabidopsis (118), rice (91) (Swaminathan et al., 2008), and soybean (143) (Ren et al., 2023), suggesting that different B3 family genes have undergone different gene duplication events. Based on phylogenetic analyses, the 70 *PgB3* genes were categorized into four subfamilies (**Figure 1**; **Supplementary Table S2**), consistent with the classification of the B3 superfamily in citrus (Liu et al., 2020), chickpea (Verma and Bhatia, 2019), tobacco (Xia et al., 2019), and *Medicago truncatula* (Gao et al., 2023). Structure and motif analyses revealed similar gene structures and motifs within each B3 gene subfamily (**Figure 2**), supporting the reliability and accuracy of the subfamily classification. Gene families are founded by a single ancestor; through duplications and deletions, the size of the family fluctuates over time, with the possibility of the family disappearing from the genome (Cannon et al., 2004). Analysis of gene duplication events and the Ka/Ks ratios of duplicated pairs (**Figure 3**, **Table 3**) indicated that the evolutionary type of all *PgB3* gene pairs was purifying selection, which further explains why pearl millet has fewer B3 family genes than other species.

### PgB3s play an important role in the stress response

Previous studies have identified important roles for B3 superfamily members in plant responses to a range of biotic and abiotic stresses. For example, oil palm ARF subfamily genes play an important role in abiotic stress (Jin et al., 2022); the wheat *REM* gene responds to cold stress (Badawi et al., 2019); overexpression of *TaRAV1* improved salt tolerance in Arabidopsis and upregulated the expression of SOS genes and other stress response genes (Luo et al., 2022). However, in this study, we found that 54 genes of the pearl millet B3 family were responsive to drought; the majority of these genes were upregulated under drought stress but a few such as *PgARF-03*, *PgREM-16*, *PgLAV-02*, and *PgRAV-04* were downregulated (**Figures 5A**, **6A**), suggesting their potential roles in the plant drought stress response. Similarly, 58 genes also responded to high-temperature stress, especially after 12h of heat treatment; many genes such as *PgLAV-02*, *PgRAV-04*, and *PgREM-20* were upregulated, indicating their involvement in regulating the plant’s response to heat stress (**Figures 5B**, **6B**). Taken together with previous findings, the variability in the functions of B3 superfamily genes under abiotic stresses is critical to plant stress responses.

### *PgRAV-04* is a negative regulator of drought stress in transgenic tobacco

Drought is a major environmental stressor that constrains plant growth and development. Plants primarily adopt two strategies to cope with drought stress: drought avoidance and enhancement of drought resistance (Basu et al., 2016). In this study, we systematically investigated the function of *PgRAV-04* in response to drought stress. Compared to the WT, the transgenic tobacco plants had a reduced ability to cope with drought stress. This conclusion was supported by the positive MDA content, the decreases in proline content and RWC, as well as the observed wilting in the leaves of transgenic plants after 16 days of water deficit. Plants defend against drought by synthesizing and accumulating osmoprotectants (including glycine, betaine, myo-inositol, and proline) to stabilize cellular substructures and maintain internal equilibrium under drought stress (Zulfiqar et al., 2020; Wang et al., 2022b). Our findings corroborate this understanding: the *PgRAV-04* overexpression plants had significantly lower proline levels than the WT (**Figure 7D**), even at 0h of drought, suggesting that the transgenic tobacco had poor drought resistance. As the drought stress intensified, the proline content remained significantly lower than the WT at 16h of drought treatment (**Figure 7D**) and the RWC was also lower (**Figure 7C**). At the same time, we observed a significant increase in MDA after 16h of drought treatment (**Figure 7B**), indicating severe cell damage under drought stress. Therefore, based on these observations, plants overexpressing *PgRAV-04* were more sensitive to drought.

In addition to combating drought through the accumulation of osmolytes, plants can also mitigate stress-induced harm through the activity of peroxidases (Minibayeva et al., 2015; Kidwai et al., 2020; Nadarajah, 2020). In this study, the activities of APX and CAT in plants overexpressing *PgRAV-04* were significantly lower than in the WT after 16 days of drought (**Figures 8A, B**); similarly, in plants overexpressing *PgRAV-04*, the activities of SOD and POD were also lower than those in the WT under severe stress conditions (**Figures 8C, D**). This further suggests that under severe drought stress, the ability of peroxidases to rescue plants is compromised. It has been reported that when stress levels increase to the point where the accumulation of reactive oxygen species exceeds the threshold of the protective enzyme system, the antioxidant capacity of plants gradually decreases (Hussain et al., 2020); this was well corroborated in our study.

Drought is a pervasive and extreme environmental challenge that poses a significant threat to plant survival and development (Chaves et al., 2003). Under conditions of water shortage, plants undergo a series of physiological and biochemical changes that not only affect their growth rate and yield (Gupta et al., 2020) but can also lead to metabolic imbalances and cellular structural damage (Joshi et al., 2016; Hussain et al., 2018). To maintain hydration and cellular homeostasis, plants initiate complex coping mechanisms that include regulating the opening and closing of stomata to reduce water loss (Basu et al., 2016), accumulating osmotic adjustment substances such as proline to maintain cell sap concentration (Hayat et al., 2012; Ghosh et al., 2022), enhancing root depth and density to improve water absorption efficiency (Vadez et al., 2013), and neutralizing reactive oxygen species produced due to water deficiency by bolstering the antioxidant defense system (Shao et al., 2007; Zhang et al., 2017; Hasanuzzaman et al., 2020; Nadarajah, 2020). In our study, overexpression of *PgRAV-04* not only diminished the drought resistance of transgenic plants through a reduction in osmolytes (proline content) (**Figure 7D**) but also decreased peroxidase activity (**Figure 8**). Among the plethora of mechanisms for stress response in plants, these two pathways in our study both contributed to the reduced drought tolerance of the transgenic plants. Therefore, we conclude that *PgRAV-04* is a negative regulator in the drought stress response.

## Conclusion

This is the first comprehensive analysis of the pearl millet B3 superfamily. We identified a total of 70 *PgB3* genes, categorized into four subfamilies. Detailed analyses were conducted on their phylogenetic relationships, exon-intron structures, chromosomal localization, gene duplication events, protein interaction network, and expression patterns under conditions of drought and high-temperature stresses. Moreover, we cloned a member of the RAV subfamily, *PgRAV-04* from pearl millet, for ectopic overexpression in tobacco and evaluated its function during drought stress. The overexpression of *PgRAV-04* increased the MDA content while decreasing proline levels and peroxidase activity, thereby enhancing the sensitivity of the transgenic plants to drought conditions. This research sets the stage for further exploration of the functional roles of the *PgB3* genes in pearl millet.

## Data availability statement

The data presented in this study were deposited in the NCBI repository, accession number PP239383. Further inquiries can be directed to the corresponding author.

## Author contributions

Y-HW: Formal analysis, Methodology, Writing – original draft, Validation. XY: Formal analysis, Methodology, Validation, Writing – original draft. B-YZ: Data curation, Investigation, Software, Writing – review & editing. W-JW: Data curation, Investigation, Software, Writing – review & editing. Z-FZ: Data curation, Investigation, Software, Writing – review & editing. X-QZ: Data curation, Formal analysis, Writing – review & editing. JD: Data curation, Formal analysis, Writing – review & editing. J-LS: Data curation, Formal analysis, Writing – review & editing. X-LH: Data curation, Writing – review & editing. K-XO: Data curation, Writing – review & editing. T-XZ: Funding acquisition, Project administration, Supervision, Writing – original draft, Writing – review & editing. F-XL: Funding acquisition, Project administration, Supervision, Writing – original draft, Writing – review & editing.
