# Supplementary material for: Comprehensive analysis of B3 family genes in pearl millet (Pennisetum glaucum) and the negative regulator role of PgRAV-04 in drought tolerance
Source: Front Plant Sci. 2024 Jul 29;15:1400301. doi: 10.3389/fpls.2024.1400301 (PMC11317251; doi:10.3389/fpls.2024.1400301)

**Supplementary data**

**Figure S1** Phylogenetic relationship of the B3 superfamilies of At (*Arabidopsis thaliana*), Os (*Oryza sativa*), and Pg (*Pennisetum glaucum*). The four colors represent four different subfamilies of the B3 family.


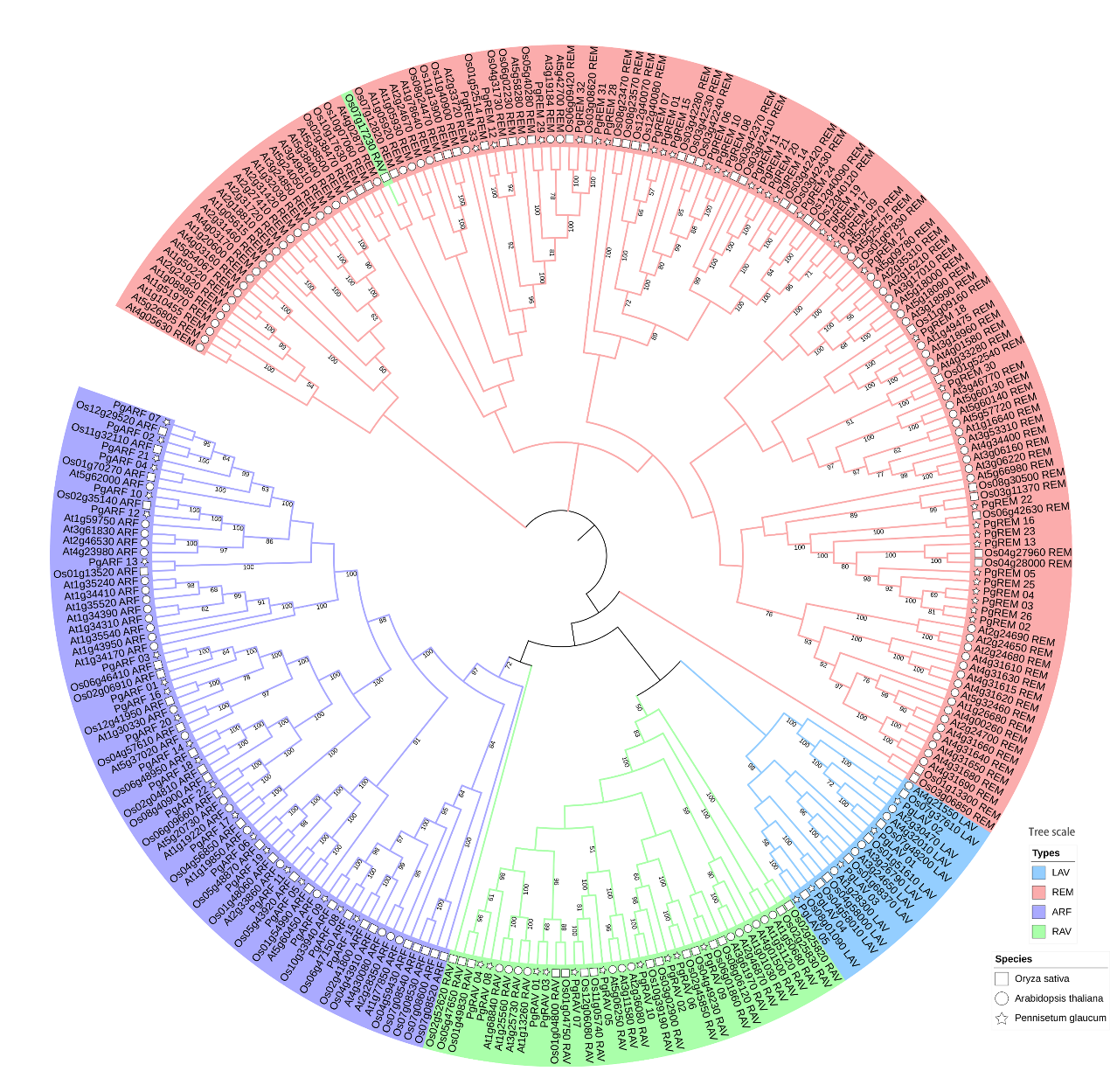


**Figure S2** Transformation process of transgenic tobacco. A: preincubate; B: induction; C: screening; D: differentiation; E: rooting.

**
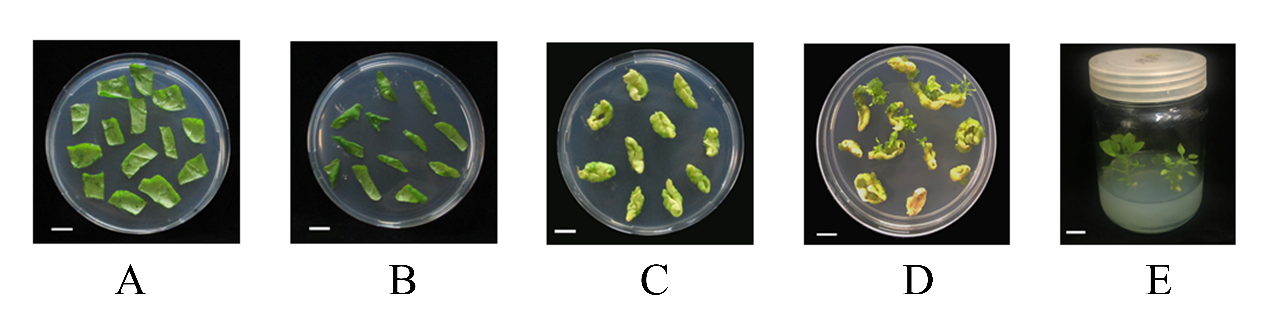
**

**Figure S3** Identification of transgenic tobacco by different techniques. A: PCR identification of DNA extracted from transgenic plants, M: DL2000 DNA Marker; 1~18 is the *PgRAV-04* OE plants; + is a positive control; − is a negative control; WT is a wild-type. B: RT-PCR identification of genetically modified tobacco，OE1-OE6 is *PgRAV-04* OE plants, WT is a wild-type. C: Real-time fluorescence quantitative PCR identification of transgenic tobacco, *Actin* was amplified as an internal reference gene. Data are mean ± SD of three replicates.


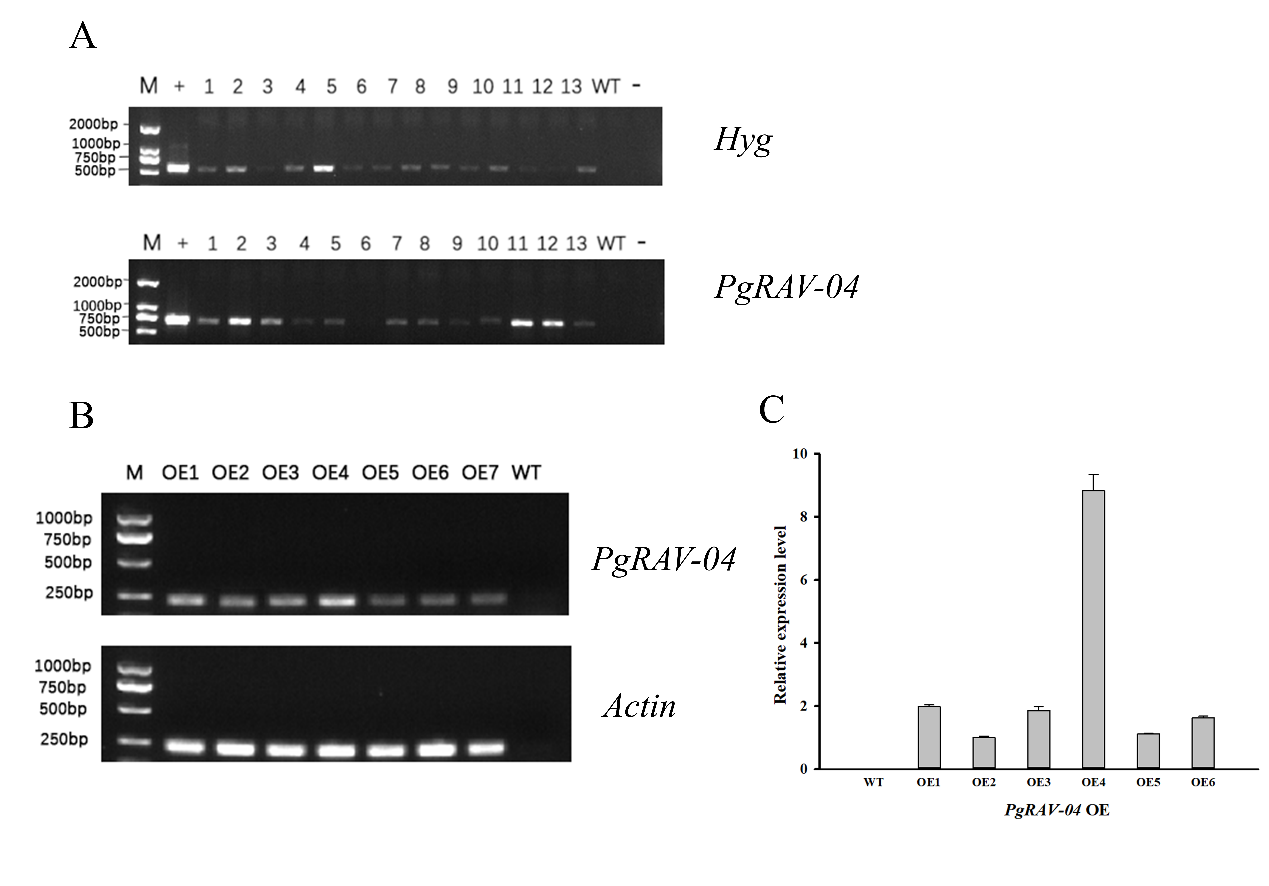

Supplement: Supplementary file 8 [file DataSheet_1.docx]
